# Population and hierarchy of active species in gold iron oxide catalysts for carbon monoxide oxidation

**DOI:** 10.1038/ncomms12905

**Published:** 2016-09-27

**Authors:** Qian He, Simon J. Freakley, Jennifer K. Edwards, Albert F. Carley, Albina Y. Borisevich, Yuki Mineo, Masatake Haruta, Graham J. Hutchings, Christopher J. Kiely

**Affiliations:** 1 Department of Materials Science and Engineering, Lehigh University, 5 East Packer Avenue, Bethlehem, Pennsylvania 18015, USA; 2 Materials Science and Technology Division, Oak Ridge National Laboratory, Oak Ridge, Tennessee 37831, USA; 3 Cardiff Catalysis Institute, School of Chemistry, Cardiff University, Main Building, Park Place, Cardiff CF10 3AT, UK; 4 Center for Nanophase Materials Sciences, Oak Ridge National Laboratory, Oak Ridge, Tennessee 37831, USA; 5 Research Center for Gold Chemistry, Graduate School of Urban Environmental Sciences, Tokyo Metropolitan University, 1-1 Minami-osawa, Hachioji, Tokyo 192-0397, Japan

## Abstract

The identity of active species in supported gold catalysts for low temperature carbon monoxide oxidation remains an unsettled debate. With large amounts of experimental evidence supporting theories of either gold nanoparticles or sub-nm gold species being active, it was recently proposed that a size-dependent activity hierarchy should exist. Here we study the diverging catalytic behaviours after heat treatment of Au/FeO_x_ materials prepared via co-precipitation and deposition precipitation methods. After ruling out any support effects, the gold particle size distributions in different catalysts are quantitatively studied using aberration corrected scanning transmission electron microscopy (STEM). A counting protocol is developed to reveal the true particle size distribution from HAADF-STEM images, which reliably includes all the gold species present. Correlation of the populations of the various gold species present with catalysis results demonstrate that a size-dependent activity hierarchy must exist in the Au/FeO_*x*_ catalyst.

## Abstract (web summary)

The identity of active species in supported gold catalysts for low-temperature carbon monoxide oxidation remains an open question. Here, the authors look at the catalytic behaviours of supported catalysts prepared under different conditions and correlate the populations of various species with activity.

## Graphical abstract

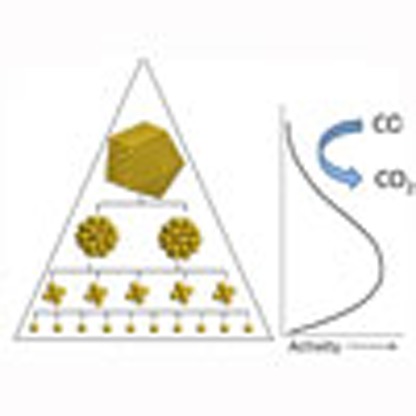

The discovery by Haruta *et al*.^1^,^2^ that CO oxidation was catalysed by Au nanoparticles supported on iron oxide has led to 30 years of scientific debate surrounding the nature of the active species^3^; however, no unequivocal identification of the active species has been reported to date and often the findings are contradictory^3^,^4^,^5^,^6^. Bond and Thompson^7^ initiated the mechanistic debate based solely on Au nanoparticles and Lopez *et al*.^8^ showed that activity increased with decreasing nanoparticle size with the optimum being 1–2 nm. Goodman *et al*.^9^,^10^ explained the role played by the Au/support periphery atoms by showing that extended Au bilayer structures on TiO_2_ were extremely active for CO oxidation. Herzing *et al*.^11^ reported a study that utilized high-angle annular dark field (HAADF) imaging in an aberration corrected STEM to investigate, for the first time, the full range of supported Au species present in real Au/FeO_x_ catalysts (namely isolated atoms, sub-nm mono- and bilayer structures and particulate species above 1 nm in size). It was found that by heat treating a highly active dried Au/FeO_*x*_ catalyst prepared by co-precipitation (CP), the number of isolated atoms observed decreased, the number of monolayer clusters remained relatively constant and the number of bilayer clusters decreased, whereas the number of nanoparticles (>1 nm) increased, all of which coincided with a measured decrease in catalytic activity. They proposed that the active catalysts contained more sub-nm bilayer clusters and fewer nanoparticles >1 nm, which agreed well with Goodman's work^9^,^10^ and also with the observations of Landman *et al*.^12^, who predicted that a minimum grouping of eight Au atoms is needed to show CO oxidation activity. More recently, Schüth *et al*.^13^ demonstrated that Au/FeO_*x*_ catalysts prepared by colloid immobilization methods can exhibit high CO oxidation activity while being devoid of any sub-nm clusters. These data combined with that of Herzing *et al*.^11^ suggest that there is not just one distinct active site for CO oxidation over supported Au species and that particles existing over a broad size range may be effective for the reaction. Such a possibility was also recently highlighted by Haruta^3^, who suggested that catalysts consist of a range of co-existing Au nanostructures each with its own characteristic activity. We propose that such an activity hierarchy might have a more general significance and readily exist in many well-studied supported metal systems, but has remained undetected and unrecognized to date because the complex diversity of metal species present, spanning a range of sizes, have not been fully characterized since aberration-corrected STEM imaging was not available to detect them in earlier studies^1^,^2^,^14^,^15^. In this work, we re-visit Au/FeO_*x*_ catalysts and use a new counting protocol to reveal the true particle size distribution (PSD) from HAADF-STEM images, which reliably includes all the Au species present and we show experimentally that an activity hierarchy does indeed exist.

## Results

### Diverging behaviour after heat treatments of two CP catalysts

**Figure 1:**
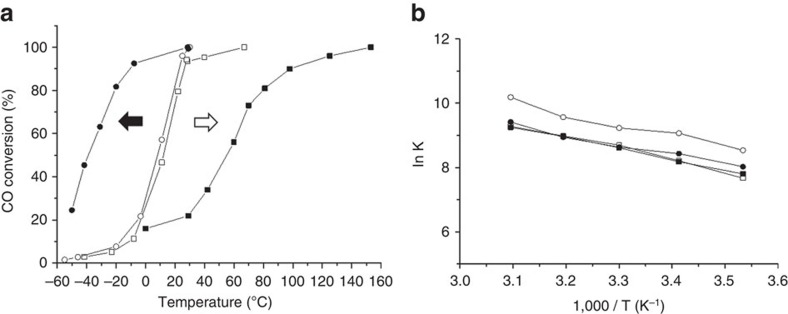
Diverging catalyst behaviour after heat treatment. (**a**) CO conversion at various temperatures. Catalyst mass 150 mg, gas flow 50 ml min^−1^ 1 vol% CO in air. (**b**) Arrhenius plots carried out at low conversion conditions. Empty circles (CP-1, dried, 6 wt% Au by ICP) filled circles (CP-1, calcined, 6 wt% Au by ICP) empty squares (CP-2, dried, 3.5 wt% Au by ICP) filled squares (CP-2, calcined, 3.5 wt% Au by ICP). The arrows shown in **a** represent the thermal activation behaviour (black arrow) of the CP-1 catalyst and the thermal deactivation behaviour (white arrow) of the CP-2 catalyst.

We studied Au/FeO_*x*_ catalysts prepared by two different co-precipitation (CP) methods previously reported by Haruta *et al*.^1^ (denoted CP-1) and Hutchings *et al*.^16^,^17^ (denoted CP-2). While the methods are similar (see ‘Methods' section for details), subtle differences exist in the sequence and rate of mixing the acidic and basic precursors. In the CP-1 method, the acidic solution (Fe(NO_3_)_3_+HAuCl_4_) was added quickly (within 2 min) into the basic solution (Na_2_CO_3_), whereas in the CP-2 method, the basic solution was slowly added drop-wise into the acidic solution over 30 min. These subtle preparation differences have dramatic effects on the catalytic behaviour. Figure 1a shows the CO oxidation activities over a range of temperatures after drying and calcination. The acid-into-base (CP-1) and base-into-acid (CP-2) catalysts at the dried-only stage (120 °C, 16 h) had similar CO conversion over the temperature range tested. However, after calcination at 300 °C for 3 h, the base-into-acid (CP-2) catalyst is deactivated, whereas the acid-into-base (CP-1) catalyst becomes more active, especially at lower temperatures as has been reported previously by Haruta^1^. This dramatic difference in activity after calcination treatment provides us with the basis sample set for this comparative structural study.

We studied the reaction kinetics to ascertain whether the higher activity at low temperature measured for the acid-into-base (CP-1) calcined sample is owing to an alternate/additional reaction pathway. Arrhenius plots were constructed for the four catalyst samples and are presented in Fig. 1b. The plot shows all of the catalysts have apparent activation energies between 25 and 30 kJ mol^−1^. The investigation of the order of reaction for CO and O_2_ (Supplementary Table 1) shows that it is first order with respect to CO and zero order with respect to O_2_ for all the catalysts. Hence, kinetic measurements strongly suggest that all four catalysts are operating by the same mechanism over the temperature range explored.

### Ruling out the possible support effect

XRD characterization (Supplementary Fig. 1a) revealed that the iron oxide support materials for the two catalysts are different. The acid-into-base (CP-1) catalysts has haematite as the predominant component. The base-into-acid (CP-2) catalysts, on the other hand, comprised a more amorphous support material identified as ferrihydrite in both the ‘dried-only' and ‘calcined' catalysts, with small contributions from haematite present in the calcined sample. It is important to investigate any potential effect the support material may have on the catalytic activities. To understand the support effect, we pre-synthesized iron oxide supports using the same two co-precipitation methods but without Au precursors, and then loaded 5% Au on them using a deposition precipitation method. These DP catalysts compared at the ‘dried-only' stage displayed equally high activity (Table 1), but after calcination, the activities of both decreased to similar levels. This demonstrates that the thermal activation behaviour observed in the acid-into-base (CP-1) catalyst was not reproduced by the corresponding DP-1 catalyst.

We then focused on investigating whether the DP-series catalysts have similar support characteristics and metal support interactions as the CP catalysts. XRD characterization (Supplementary Fig. 1b) showed that the desired haematite (DP-1) and ferrihydrite (DP-2) phases were obtained. The surface area of the DP catalysts was found to be very similar to the corresponding CP catalysts, determined using nitrogen adsorption and BET isotherm analysis (Supplementary Table 2). These experiments confirmed that the desired support materials were indeed obtained in the DP catalysts.

We further investigated the interaction between Au and the support material, which has been suggested to have a significant impact on the catalytic properties. XPS (X-ray photoemission spectroscopy) characterization was carried out for CP-1 and CP-2 catalysts, in both the ‘dried-only' and ‘calcined' state. As shown in Supplementary Fig. 2, only metallic Au^0^ species were found, suggesting that Au interacts similarly with the two different support materials, as cationic Au species were absent or below the detection limit. In addition, temperature programmed reduction (TPR) experiments were carried out on the bare catalyst supports and the corresponding CP and DP catalysts. As shown in Supplementary Fig. 3, the presence of Au has a substantial effect on the TPR profile of the iron oxides, shifting reduction peaks to a much lower temperature in all cases. However, the exact nature of such shifts could be the result of many factors in such complex catalyst systems. It could be due to (i) the reduction of Au species^18^ (ii) an easier decomposition of Fe-hydroxide species due to electronic interaction between Au and iron oxides^19^,^20^, (iii) a Au-catalysed hydrogenation reaction^21^ or (iv) some combination of these effects The magnitude of the shift and change of the peak shapes are also sensitive to parameters such as Au loading^22^. Nevertheless, we did observe a similar TPR behaviour between the CP-2 and the DP-2 catalysts, suggesting that the the metal/support interaction in these catalysts bear some resemblance to each other. Therefore, a simple support effect cannot be responsible for the varying thermal activation/deactivation behaviours observed in these catalysts on heat treatments.

### PSD statistics

**Figure 2:**
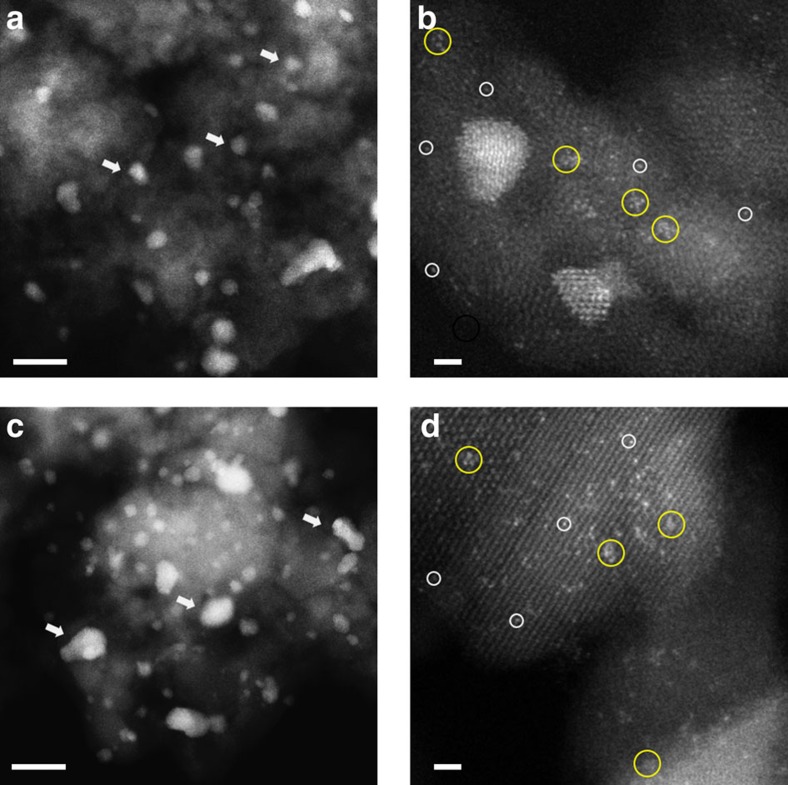
Representative HAADF-STEM images of CP-1 acid into base catalysts. Images from ‘dried-only' (**a**,**b**) and ‘calcined' catalysts (**c**,**d**) showed the co-existence of nanoparticles of various sizes, sub-nm clusters and isolated atoms. Au nanoparticles—white arrows; sub-nm Au clusters—yellow circles; and isolated Au atoms—white circles. Scale bars in **a**,**c** represent 10 nm. Scale bars in **b**,**d** represent 1 nm.

**Figure 3:**
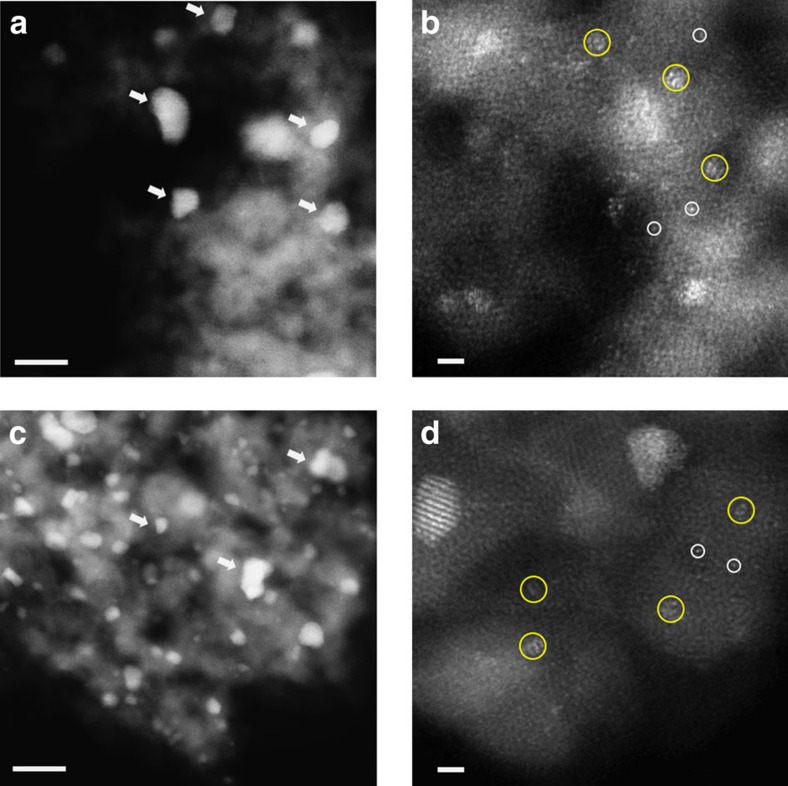
Representative HAADF-STEM images of CP-2 base into acid catalysts. Images from ‘dried-only' (**a**,**b**) and ‘calcined' catalysts (**c**,**d**) showed the co-existence of nanoparticles of various sizes, sub-nm clusters and isolated atoms. Au nanoparticles—white arrows; sub-nm Au clusters—yellow circles; and isolated Au atoms—white circles. Scale bars in **a**,**c** represent 10 nm. Scale bars in **b**,**d** represent 1 nm.

**Figure 4:**
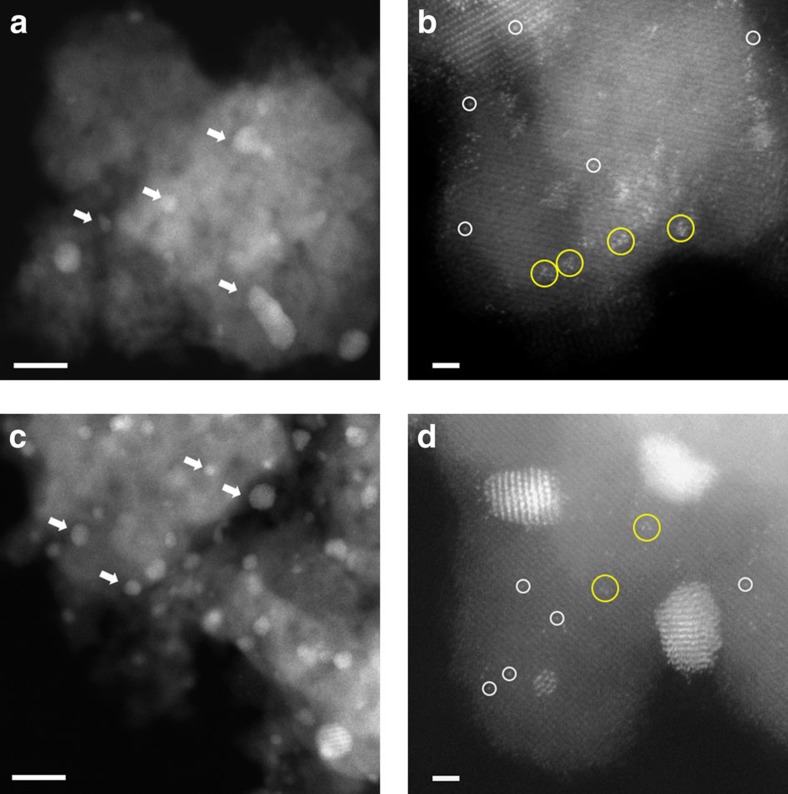
Representative HAADF-STEM images of DP-1 acid into base catalysts. Images from ‘dried-only' (**a**,**b**) and ‘calcined' catalysts (**c**,**d**) showed the co-existence of nanoparticles of various sizes, sub-nm clusters and isolated atoms. Au nanoparticles—white arrows; sub-nm Au clusters—yellow circles; and isolated Au atoms—white circles. Scale bars in **a**,**c** represent 10 nm. Scale bars in **b**,**d** represent 1 nm.

Having ruled out the support effect from the above arguments, we concluded that the different catalytic activities observed are very likely owing to the different relative populations of active Au species present. To identify and quantify the active Au species, the catalysts were characterized by aberration-corrected HAADF-STEM imaging. The sub-Å spatial resolution and high sensitivity of incoherent scattering to atomic number associated with the HAADF-STEM imaging mode allows for the efficient detection of even single Au atoms dispersed on or in FeO_*x*_ supports^11^,^23^. Representative images are shown for the catalysts prepared by co-precipitation (CP-1 in Fig. 2; CP-2 in Fig. 3) and deposition-precipitation (DP-1 in Fig. 4). For each, representative lower and higher magnification HAADF images are shown, displaying Au nanoparticles, sub-nm clusters and atomically dispersed species. Although co-existence of these Au-species has been previously demonstrated for CP-type catalysts, it has not previously been recognized that DP-type Au/FeO_*x*_ catalysts can contain sub-nm clusters and atomically dispersed Au that are not readily visible by phase contrast TEM imaging.

The next challenge was to quantify the PSDs for these catalysts. Conventionally, this is done by measuring the number frequency of particles falling within certain size intervals from experimental images. However, this approach is not suitable here because it does not ensure a fair/sufficient sampling of Au species owing to their size differences (spanning the 0.2–20.0 nm range) and consequently their different visibilities in the microscope under specific imaging conditions. For instance, sub-nm Au and atomically dispersed species can be easily under-sampled as they are visible only at the highest magnification (e.g. × 10,000,000 with a field of view 8 nm × 8 nm) and only in areas where the support material is thin enough to generate sufficient contrast from the Au atoms. Conversely, such a small field of view in a high magnification image rarely contains enough of the larger Au nanoparticles to be statistically relevant. In addition, PSD comparisons based on number frequencies overlook the fact that the total number of particles in the catalyst is not known and can differ greatly from sample to sample. We have therefore developed a new systematic particle analysis protocol in an attempt to overcome these problems. First, particle counts are normalized by the projected area of the support material to get the particle number density and ensure fair/sufficient sampling of all the Au species present despite their differing size scales and varying visibilities. In practice, Au species above 1 nm were counted from lower (× 1,000,000) magnification images and sub-nm clusters and isolated atoms were counted from higher (× 10,000,000) magnification images. A detailed explanation of the image processing procedures used for particle counting is given in Supplementary Fig. 4 and Supplementary Notes 1. In the next step, experimentally determined number density values are converted into mass fractions (that is, the fraction of total Au atoms in the sample that end up in the particles within a specific size range), which in principle allows meaningful comparisons between different samples with known Au loading as measured by an independent technique such as ICP or AAS.

Practically, the number of Au atoms contained in nanoparticles of a certain size can be estimated from geometrical considerations for example, using a hemispherical Mackay icosahedral model^24^, (see Supplementary Table 3). Similar geometric arguments are used to estimate the number of exposed surface atoms associated with each particle and hence the surface fraction (that is, the fraction of total Au atoms in the sample that end up on the surface of particles within a specific size range). Furthermore, the number of Au atoms at the particle/FeO_*x*_ interface associated with each particle can also be estimated allowing us to deduce the Au periphery atom fraction (that is, the fraction of total Au atoms in the sample that end up on the interfacial periphery of particles within a specific size range). However, it should be noted that geometrical estimates become increasingly inaccurate (especially for the first two parameters) because as the particle radius *r* gets larger, the total number of atoms per particle scales as *r*^3^, while the number of surface atoms varies as *r*^2^ and the perimeter length scales as *r*. Thus an artificial cut-off was applied in our analysis such that particles larger than 7 nm in diameter were neglected, because (i) larger particles are widely believed to be catalytically irrelevant^8^; and (ii) Au particles larger than 7 nm are rarely observed and give extremely low number densities with high uncertainty (Supplementary Fig. 5 gives further justification of this cut-off procedure). However, neglecting occasional large Au particles removes the basis for comparison among different catalyst samples because, although low in number density, larger particles consume a significant amount of total Au mass (for example, the mass of one 10 nm particle is approximately equivalent to a thousand 1 nm particles). Fortunately, assuming these larger particles remain relatively unchanged during heating, a fair comparison can still be achieved for the same catalyst material at different stages of heat treatment. This assumption is supported by the classic work of Buffat *et al*.^25^ showing that larger Au particles have a significantly higher melting temperature than smaller particles, and the recent microscopy observations by Allard *et al*.^26^,^27^ on the size evolution of Au species during *in situ* heating.

**Figure 5:**
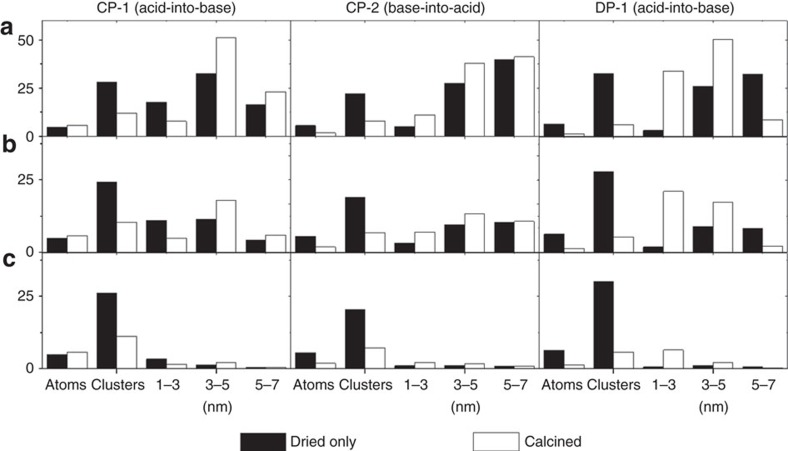
Particle size distribution of various Au species in the catalysts. Mass fraction (**a**), surface fractions (**b**) and perimeter fractions (**c**) of different Au species for the CP-1, CP-2 and DP-1 catalysts, calculated from micrographs such as those presented in Figs 2c,d; 3c,d and 4c,d. The filled and empty bars represent the ‘dried-only' and ‘calcined' states of the catalyst, respectively.

The resultant raw number density PSDs for the CP-1, CP-2 and DP catalysts at the ‘dried-only' and ‘calcined' stages are shown in Supplementary Fig. 5. These histograms for the CP-1, CP-2 and DP-1 materials are re-plotted in Fig. 5 in terms of Au mass fraction (top row), Au surface atom fraction (middle row) and interfacial periphery atom fraction (bottom row) for the Au atoms, sub-nm clusters and Au nanoparticles in the 1–3 nm, 3–5 nm and 5–7 nm size intervals, respectively. This type of data presentation is more convenient for correlating the Au species population within each sample to its overall measured catalytic performance.

## Discussion

Based on the evidence, it is not possible to assign just one type of Au species as being solely active, while the others are inactive, to explain all three sets of data. For instance, if the 1–3 nm Au particles are the only active species, then the thermal deactivation of the DP-1 catalyst cannot be explained since a larger population of these 1–3 nm particles exists after calcination. Conversely, if the sub-nm Au clusters are the only active species, then the thermal activation behaviour of the acid-into-base (CP-1) catalysts cannot be explained as there is a smaller population of these sub-nm Au clusters after calcination. Several PSDs derived from the same data set using different cut-off values and binning sizes are shown in Supplementary Fig. 6 where similar trends were observed; neither sub-nm clusters nor nanoparticles can be the only active site in this catalyst system as is often reported.

Instead we argue that an activity hierarchy for the different Au species present must exist to explain the observed behaviour (that is, the co-existence of wide range of Au nanostructures each having a different intrinsic activity needs to be considered). Hence the final reported activities of the catalysts should be a weighted sum of the activity of each of the different species present, combined with their relative population densities (that is, total activity *A*=∑_i_*ρ*_i_*ɛ*_i_, where *ρ*_i_ and *ɛ*_i_ represent the population fraction and intrinsic activity for the i-th active species). The relative activities *ɛ*_i_ of different Au species have previously been estimated by Haruta^3^ based on a literature survey from sources describing Au species supported on different model oxide supports. Here we provide conclusive experimental evidence that such an activity hierarchy actually exists in the ‘real' Au/FeO_*x*_ catalyst system.

To fully quantify the activity of every Au species present, we would need to acquire systematic PSD and activity data from a catalyst calcined at many different temperatures. In this work, we only have two experimentally determined overall activity data points (that is, from the ‘dried-only' and ‘calcined at 300 °C' version of each sample) and the corresponding species population fraction *ρ*_i_ measurements from these samples. Therefore, we necessarily need to assume that only two species are dominating the observed catalytic behaviour to solve for their *ɛ*_i_ values. Prior reports exist demonstrating that catalysts consisting of isolated Au atoms have a low intrinsic activity for CO oxidation; however, compared with the current study, the activity is typically an order of magnitude lower per surface Au atom compared with the particles and clusters present in the DP calcined catalyst^28^. This is consistent with the observations of Gates and co-workers who demonstrated that CO oxidation activity significantly increased as atomic Au species sintered to form sub-nm Au clusters^29^. Lopez *et al*.^8^ also suggest that activity is greatly reduced when the average particle size becomes larger than 5–7 nm. On the basis of this, we conclude that atomic Au species and particle above 5 nm are unlikely to be highly active species for low temperature CO oxidation. Hence sub-nm clusters^11^ and 1–3 nm particles^13^ were chosen as the most likely highest-activity candidates as they have both been proposed as the single most-active entity in prior literature. On the basis of the analysis of the two activity data points for ‘dried-only' and ‘calcined' states of the deposition precipitated (DP-1) catalyst, combined with the amount of Au surface available for each measured particle size, we estimate the activity to be ∼0.42 and ∼0.21 mol_CO_ per mol_surface Au atoms_ per second for the sub-nm clusters and 1–3 nm particles, respectively. If we consider that only gold atoms on the periphery sites are active, the estimated activity is ∼0.40 and ∼0.60 mol_CO_ per mol_peripheral Au atoms_ per second for the sub-nm clusters and 1–3 nm particles, respectively (For details, see Supplementary Tables 4 and 5 and Supplementary Notes 2). Interestingly, these activity values are very similar to those previously estimated by Haruta for sub-nm clusters and 1–3 nm nanoparticles^3^. To within the limits of experimental error and the approximations incurred by our assumptions, this implies that the surface, irrespective of whether it exists on a nanoparticle or sub-nm cluster, exhibits a similar intrinsic activity, and presumably facilitates the same Bond–Thompson reaction mechanism^7^. However, it should be noted that the observed catalytic activity would be dominated by the sub-nm clusters in the Au/FeO_x_ system owing to the much higher population of these species relative to 1–3 nm Au particles. It is also implicit that producing many sub-nm clusters containing more than eight atoms, as opposed to generating fewer larger nanoparticles is the most efficient way of utilizing the gold.

The overall catalytic activity and Au species population measurements on the co-precipitated acid-into-base (CP-1) and base-into-acid (CP-2) catalysts could not be analysed in an analogous fashion because of the high probability that a significant fraction of the ultra-small Au species are actually embedded inside the support material during the co-precipitation technique, which would not be exposed to the reactants, but would be impossible to exclude from the PSD measurements. This is not an issue for the DP-1 catalyst analysed above as the haematite support material is pre-formed before the Au precursor is added ensuring that all the atomic and cluster species exist solely as surface entities.

The ‘anomalous' thermal activation behaviour observed for the acid-into-base (CP-1) catalysts, as opposed to deactivation for the corresponding base-into-acid (CP-2) catalyst, can now be explained via a comparison of their thermal evolution process^26^,^27^. It is probable that during the preparation of the CP-1 catalyst, the quicker mixing of the precursors results in a more homogenous precipitation, which, in turn, enhances the population of Au species embedded within the oxide support (that is, the structure is effectively in a non-equilibrium state). This embedding effect has been verified experimentally by taking a sequence of images where the convergent probe was focused at different depths through the iron oxide particles to reveal the height location of Au species dispersed through the depth of the iron oxide particles (see Supplementary Fig. 7). It should also be noted that images captured before and after through focal series acquisition (Supplementary Fig. 8) show minimal change in the population densities of atoms, clusters and nanoparticles in the area irradiated, proving that our population density measurements are not affected by electron beam irradiation under the imaging conditions used.

**Figure 6:**
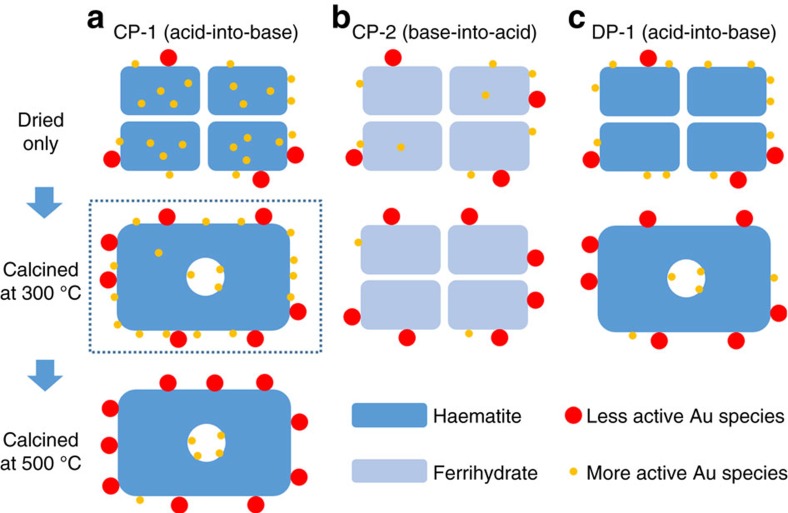
Proposed mechanism for the thermal activation behaviour of the CP-1 catalyst. A series of schematic diagrams that illustrate the thermal evolution process of the (**a**) CP-1 (acid-into-base), (**b**) CP-2 (base-into-acid) and (**c**) DP-1(acid-into-base) catalysts. The CP-1 catalyst (column **a**) has a much larger amount of atomic Au species buried inside the support material after only being dried as compared with the CP-2 and DP-1 catalysts (columns **b**,**c**, respectively). Therefore, after calcination at 300 °C, the loss of the more active smaller Au species (that is, sub-nm clusters and 1–3 nm Au particles) owing to agglomeration can be replenished in the CP-1 catalysts by the outward diffusion of the ‘trapped' internal Au species, which is not possible in the case of the CP-2 and DP-1 catalysts. As a result, the CP-1 catalyst after calcination at 300 °C can be even more active than the dried-only stage (as highlighted by the dashed box). However, after prolonged calcination at higher temperatures (that is, 500 °C), the Au reserves inside the CP-1 support particle eventually get depleted and the catalytic activity drops owing to agglomeration of the surface Au species.

The probable microstructural evolution sequence for the CP-1, CP-2 and DP-1 catalysts is compared schematically in Fig. 6. During heat treatment, the smallest Au-species (dispersed atoms and sub-nm clusters) originally on the surface will have a tendency to diffuse and agglomerate into larger particles, whereas Au species trapped within the support migrate towards the surface during heat treatment and replenish active Au species. The net result on catalytic activity will depend on the balance between the rates of agglomeration of existing surface species and the rate of replenishment process of these small active Au species from the bulk. For the CP-1 catalyst, it is probable that the replenishment process via migration dominates over the 3 h calcination period, resulting in an overall increase in the number density exposed active Au species. Interestingly, when the CP-1 catalyst was calcined to 500 °C for 3 h, the CO conversion at 25 °C effectively decreased to ∼0%. This behaviour can also be explained if there are no longer enough embedded Au species in the sub-surface reservoir to compensate the loss of active Au species through accelerated sintering and migration at this higher calcination temperature. The proposed migration of Au from sub-surface to surface positions in FeO_*x*_ support particles during calcination has already been demonstrated by the elegant *in situ* electron microscopy experiments of Allard *et al*.^26^,^27^ By way of comparison, the CP-2 catalyst does not have such an abundant reservoir of sub-surface gold species to replenish the lost sub-nm clusters during calcination, because during the preparation the basic precursor was added into acidic precursor slowly thus decreasing chance of Au being trapped inside the oxide support (that is, the material is already much closer to being in an equilibrium state). Hence this CP-2 sample simply deactivated on heat treatment. The same is true for the DP catalysts, where the Au was added after the support material was formed, which means that the proposed mechanism of activation upon thermal treatment cannot be invoked.

In summary, Au/FeO_*x*_ catalysts were prepared using two different co-precipitation methods. The acid-into-base (CP-1) catalysts showed a unique thermal activation behaviour, whereas the base-into-acid (CP-2) catalysts deactivated after the same calcination treatment. Additional catalytic data from a comparable set of deposition precipitated (DP) catalysts show that the diverging behaviour is not owing to either a different reaction mechanism or a support effect. Detailed PSD measurements on these samples using a new data analysis protocol for HAADF-STEM images, allowed us to identify an activity hierarchy with respect to the various supported Au species that co-exist. The most active species, namely sub-nm clusters and 1–3 nm Au particles were estimated to have intrinsic activities of 0.42 and ∼0.21 mol_CO_ per mol_surface Au atoms_ per second, respectively. Furthermore, the observed activity in our Au/FeO_*x*_ samples are dominated by the sub-nm clusters by virtue of their extremely high population density relative to 1–3 nm particles. The anomalous thermal activation behaviour displayed by the acid-into-base CP-1 catalyst arises because its synthesis procedure improves the chances of having Au species embedded within the FeO_*x*_ support that can subsequently serve to replenish the dwindling population of highly active sub-nm Au clusters during calcination.

This study brings new insight into our understanding of supported Au catalysts for low temperature CO oxidation and provides new direction towards settling the ongoing debate over the identity of the active Au species. We postulate that the activity of this well-studied system, and most likely many other supported catalyst systems, are the result of a complex population versus activity hierarchy relationship where the measured overall catalytic activity needs to be thought of as a weighted sum of the activity of each of the different species present combined with their relative population densities (that is, total activity *A*=∑_i_*ρ*_i_*ɛ*_i_, where *ρ*_i_ and *ɛ*_i_ represent the population fraction and intrinsic activity for the i-th active species). We also provide a new experimental protocol for measuring the population densities of all the supported species present in such a catalyst system in a statistically relevant manner.

## Methods

### Sample preparation

Co-precipitated (CP-1 series) catalysts. Gold supported on iron oxide was prepared by the co-precipitation of HAuCl_4_ and Fe(NO_3_)_3_.9H_2_O using Na_2_CO_3_. A typical preparation of 5 wt% (nominal) Au/Fe_2_O_3_ was carried out according to the following procedure. The acidic HAuCl_4_ and Fe(NO_3_)_3_.9H_2_O solution was added quickly (that is, within 2 min) into the basic Na_2_CO_3_ solution while the solution was heated to 60 °C. The final pH was also fixed at 8.5 and the solution was left to age for 1 h under conditions of continuous stirring. The resultant solution was then centrifuged with hot water at around 80 °C. The solid retrieved in this way was then dried in a GC oven under flowing air at 120 °C for 16 h. A portion of the sample was then calcined at 300 °C for 3 h using a ramp rate of 20 °C min^−1^.

Co-precipitated (CP-2 series) catalysts. A second set of gold supported on iron oxide catalysts was prepared by a co-precipitation method that differed in the way of mixing acidic precursors with the basic precursors. A typical preparation of 5 wt% Au/Fe_2_O_3_ was carried out according to the following procedure. Fe(NO_3_).9H_2_O (4.807 g, 99.95%, Sigma-Aldrich) was dissolved in 400 ml of deionized water, HAuCl_4_ (4.80 ml, 12.25 g Au in 1,000 ml, 99.9%, Aldrich) was added to this solution and stirred vigorously while the solution was heated to 80 °C. The pH of the solution was increased by adding Na_2_CO_3_ drop-wise over a period of 30 min until the pH reached 8.5 and it was then left for 1 h under continuous stirring conditions. The solution was then filtered and washed with 2 l of deionized water. The solid was then dried at 120 °C for 16 h. Part of the sample was then calcined at 300 °C for 3 h with a ramp rate of 20 °C min^−1^ under static air.

Deposition precipitation (DP-1, DP-2 series) catalysts. Gold supported on iron oxide was also prepared by a deposition precipitation method. A typical preparation of 1 g of 5 wt% (nominal) Au/iron oxide is outlined by the following procedure. An amount 0.95 g of a pre-prepared iron oxide (produced by CP-1 and CP-2 methods without the addition of Au) support was suspended in 400 ml of deionized water and held at 60 °C under vigorous stirring. HAuCl_4_ (4.80 ml, 12.25 g Au in 1,000 ml, 99.9%, Aldrich) was added to this solution and stirred for 15 min. Na_2_CO_3_ solution was then added drop-wise until the pH of the solution stabilized at 8.5. The preparation was left for 1 h before filtration and washing with 2 l of hot deionized water (80 °C). The sample was then dried at 110 °C for 16 h followed by calcination at 300 °C for 3 h with a ramp rate of 20 °C min^−1^ under static air.

### Catalyst testing

Test Method 1. Au/Fe_2_O_3_ catalysts were tested for CO oxidation using a glass micro-reactor with internal diameter 0.5 cm. Typically 10 mg of catalyst was packed between two small pieces of glass wool fixed inside a thermostatic water bath that was held at 25 °C for the duration of the reaction. The gas feed, 5,000 p.p.m. CO in synthetic air, was passed through the catalyst bed at various flow rates (25–100 ml min^−1^). The reaction products were analysed by Varian 3800 on-line GC with a 1.5 m Carbosieve column.

Test Method 2. A similar, but more flexible, catalytic test set-up was used, which allows the reaction temperature and reactor concentrations to be varied, therefore enabling reaction kinetics to be studied. Typically, between 10 and 50 mg of catalyst was diluted with between 190 and 150 mg of alumina to give a total sample mass of 200 mg. This mixture was packed between two pieces of glass wool in a 6 mm diameter u-shaped glass reactor. Using three flow controllers to mix gases, it was possible to use 0.5–10 vol% CO and 1–20 vol% O_2_ at flow rates of between 10 and 100 ml min^−1^, with the balance of the gas being made up by He. Using water and oil baths, it was possible to carry out the reactions at temperatures between 10 and 120 °C. A cooling unit was also added to achieve reaction temperatures below 0 °C.

### Catalyst characterization

BET surface area analysis was determined using a Micromeritics Gemini 2360 analyser. A known amount of sample, typically between 100 and 200 mg, was placed in a straight-walled tube and degassed for 1 h at 120 °C under a flow of N_2_. The surface area was analysed using a single-point analysis typically taking five points between *P*/*P*_o_=0.05–0.1.

X-ray diffraction of the catalyst materials was carried out using a PANalytical X'pert Pro powder diffractometer using a Cu Kα radiation source operating at 40 kV and 40 mA. Standard analysis was performed using a 40 min scan between 2*θ* values of 10–80° with the samples supported on an amorphous silicon wafer. The phases were identified from the diffraction patterns using the ICDD database.

XPS measurements performed using a Kratos Axis Ultra-DLD photoelectron spectrometer using a monochromatic Al K_α_ source operating at 144 W (10 mA × 14 kV emission). The analysis was performed in the hybrid mode affording greater sensitivity, over a sample area of ∼210 μm^2^ at pass energies of 40 and 160 eV for high resolution and survey scans, respectively. Charge compensation was achieved using an immersion lens system and all the data were subsequently calibrated to the C(1s) core level at 284.5 eV. All binding energies are quoted with an accuracy of ±0.2 eV.

TPR was carried using a ChemBet TPR/TPD equipped with a TCD detector. Each analysis was carried out with 0.1 g of catalyst, which was pre-treated at 110 °C for 1 h under a He flow of 100 ml min^−1^. TPR was carried out using a 10 % H_2_/Ar mixture at a flow rate of 100 ml min^−1^ and by heating from room temperature to 800 °C at a ramp rate of 10 °C min^−1^.

Samples for examination by STEM were prepared by dry dispersing the catalyst powder onto a holey carbon supported by a 300-mesh copper TEM grid (SPI supplies). The HAADF-STEM images were taken using the aberration-corrected JEOL 2200FS transmission electron microscope at Lehigh University and a Nion UltraSTEM 100 and 200 at Oak Ridge National Laboratory. The PSD analysis was performed using Image J.

ICP-OES was carried out by an external company, Warwick Analytical Services, under ISO 17025 accreditation.

### Data availability

The data that support the findings of this study are available from the corresponding authors upon request.

## Additional information

**How to cite this article:** He, Q. *et al*. Population and hierarchy of active species in gold iron oxide catalysts for carbon monoxide oxidation. *Nat. Commun.* 7:12905 doi: 10.1038/ncomms12905 (2016).

## Supplementary Material

Supplementary Information Supplementary Figures 1-8, Supplementary Tables 1-5, Supplementary Notes 1-2 and Supplementary References

## Figures and Tables

**Table 1:** CO conversion of catalysts prepared by DP.

Catalyst	Dried 120 °C per 16 h	Calcined 300 °C per 3 h
DP-1 (acid-into-base)	92%	49%
DP-2 (base-into-acid)	89%	47%

CO conversion over 5 wt% Au/FeO_*x*_ prepared by a deposition precipitation method on haematite (DP-1) and ferrihydrite (DP-2) supports that were dried (110 °C, 16 h) and then calcined (300 °C for 3 h).

Testing conditions: 0.5 vol% CO/air, 100 ml min^−1^, 10 mg catalyst, 25 °C.
