# Supplementary material for: Population and hierarchy of active species in gold iron oxide catalysts for carbon monoxide oxidation
Source: Nat Commun. 2016 Sep 27;7:12905. doi: 10.1038/ncomms12905 (PMC5052626; doi:10.1038/ncomms12905)
Supplement: Supplementary Information — Supplementary Figures 1-8, Supplementary Tables 1-5, Supplementary Notes 1-2 and Supplementary References [file ncomms12905-s1.pdf]

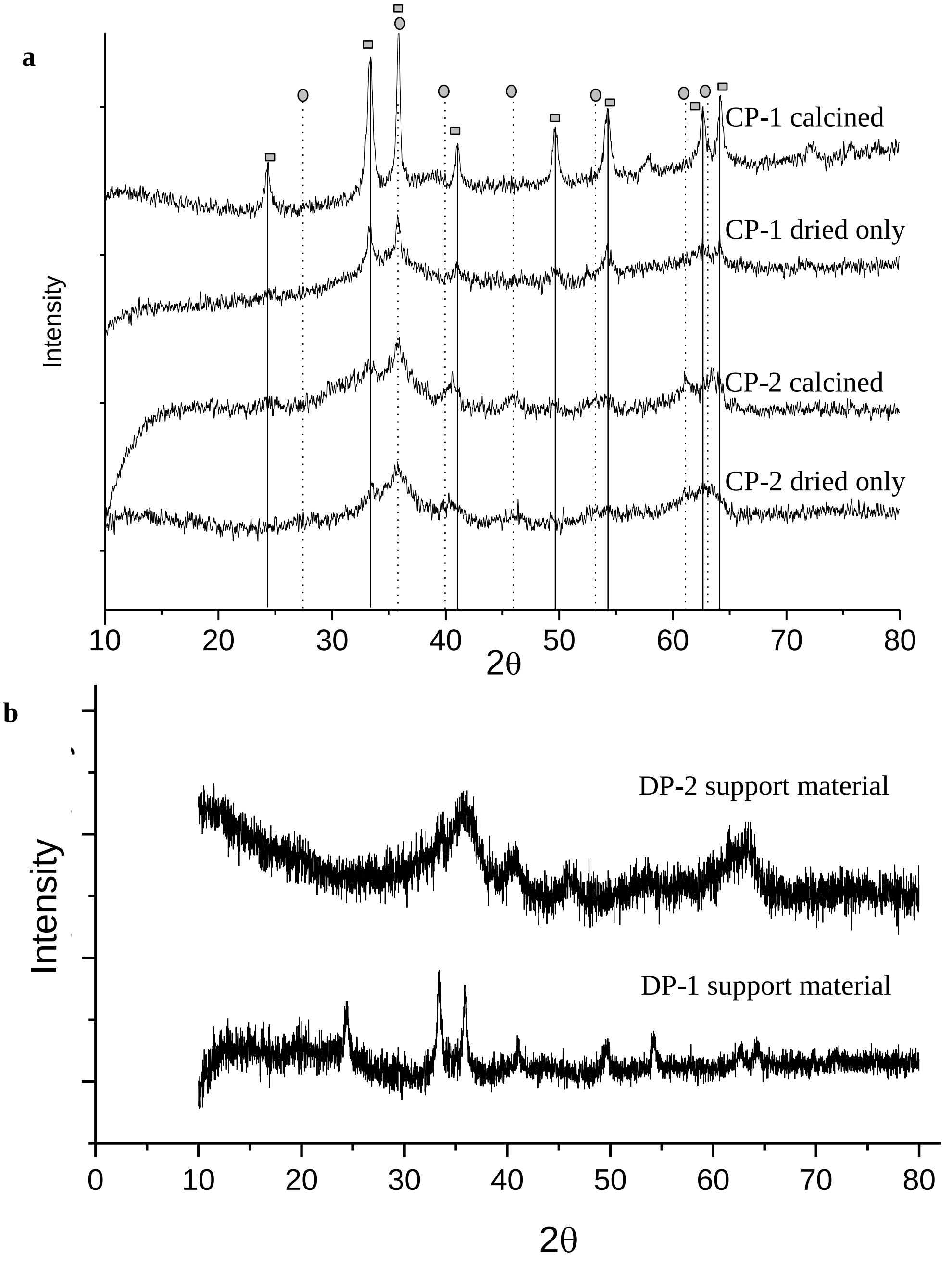

Supplementary Figure 1. Results from XRD measurements. a X-ray diffraction patterns of CP-1 (acid-into-base) and CP-2 (base-into-acid) catalysts at the 'dried only' and 'calcined' stages. Haematite (Fe_2O_3) peaks are indicated by filled squares ■ and ferrihydrite ($Fe_2O_3 \cdot 0.5\ H_2O$) peaks by filled circles ●. **b** X-ray diffraction patterns of DP-1 and DP-2 support materials

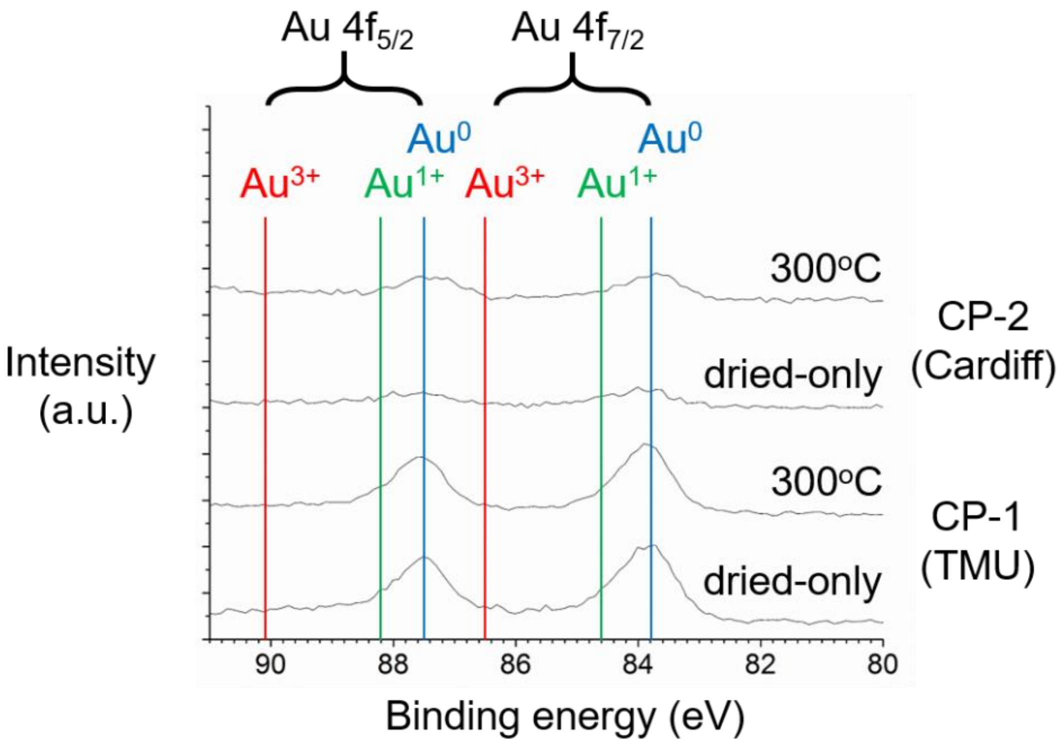

Supplementary Figure 2. Results from XPS measurements. X-ray photoelectron Au 4f spectra of the CP-1 and CP-2 catalysts at the 'dried-only' and 'calcined' (300°C) stages. The reference peak positions of Au having different oxidation states was obtained from Reference [1] and are marked: Au^0 – blue, 83.8/87.5eV, Au^{1+} – green, 84.6/88.2 eV and Au^{3+} – red, 86.5/90.1 eV. The Au species present in all catalysts are seen to be predominantly metallic.

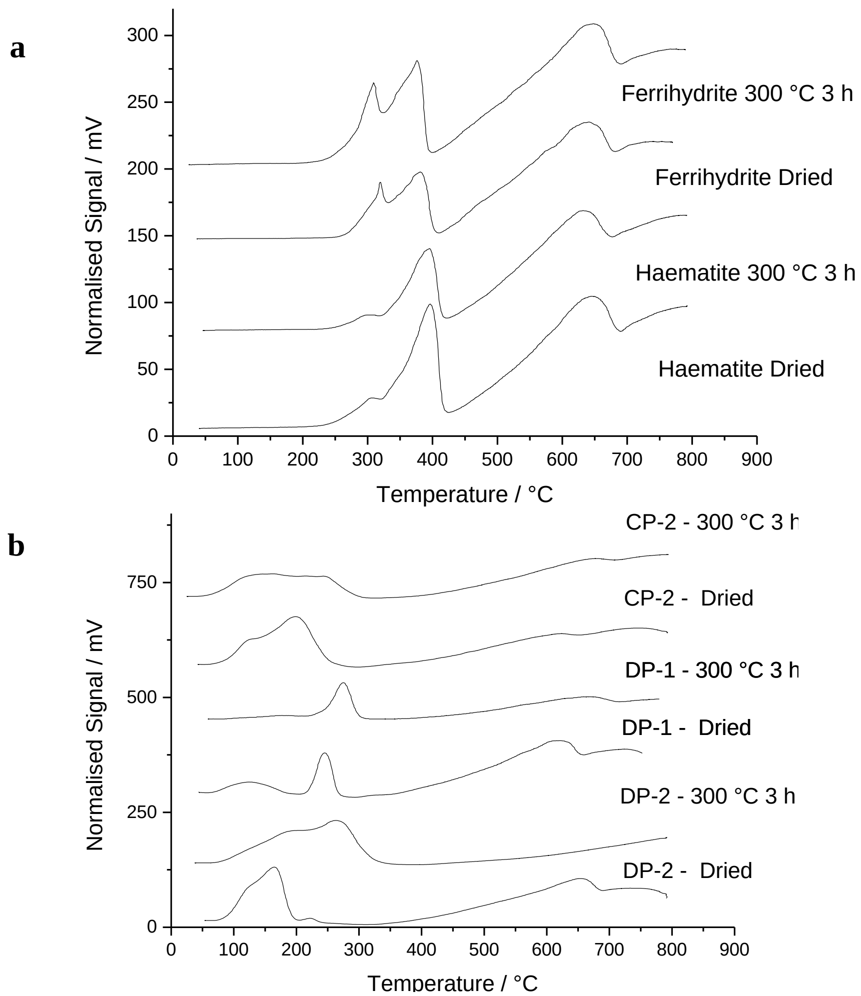

Supplementary Figure 3. Results from TPR measurements. a TPR measurements of bare haematite and ferrihydrite support materials after drying (110 °C, 16 h) and calcination (300°C, 3 h, 20 °C min^{-1}) and **b** corresponding TPR measurements on supported gold CP-2 (ferrihydrite), DP-1 (haematite) and DP-2 (ferrihydrite) catalysts after identical heat treatments to those specified in **a**.

The TPR profiles in the figures above show features that are broadly in line with the literature on the reduction of iron oxides.[2] The initial reduction feature at ~ 300°C can be assigned to the decomposition of Fe-hydroxide features to generate haematite (Fe_2O_3). The second feature present at ~380 °C corresponds to the haematite to magnetite (Fe_3O_4) reduction followed by a broad reduction feature corresponding to the reduction of magnetite through FeO towards metallic Fe. In the corresponding TPRs of the catalyst materials (Supplementary Figure 3 **b**) a clear shift in the temperature of the haematite to magnetite reduction peak can be observed for all the gold containing materials while the high temperature reduction of magnetite remains largely unaffected. The reduction of the temperatures of haematite reduction. However, the exact nature of such shifts could be the result of many factors in such complex catalyst systems. It could be due to (i) the reduction of Au species[2] (ii) an easier decomposition of Fe-hydroxide species due to electronic interaction between Au and iron oxides,[3,4] (iii) a Au catalysed hydrogenation reaction[5] or (iv) some combination of these effects The magnitude of the shift and change of the peak shapes are also sensitive to parameters such as Au loading.[6]

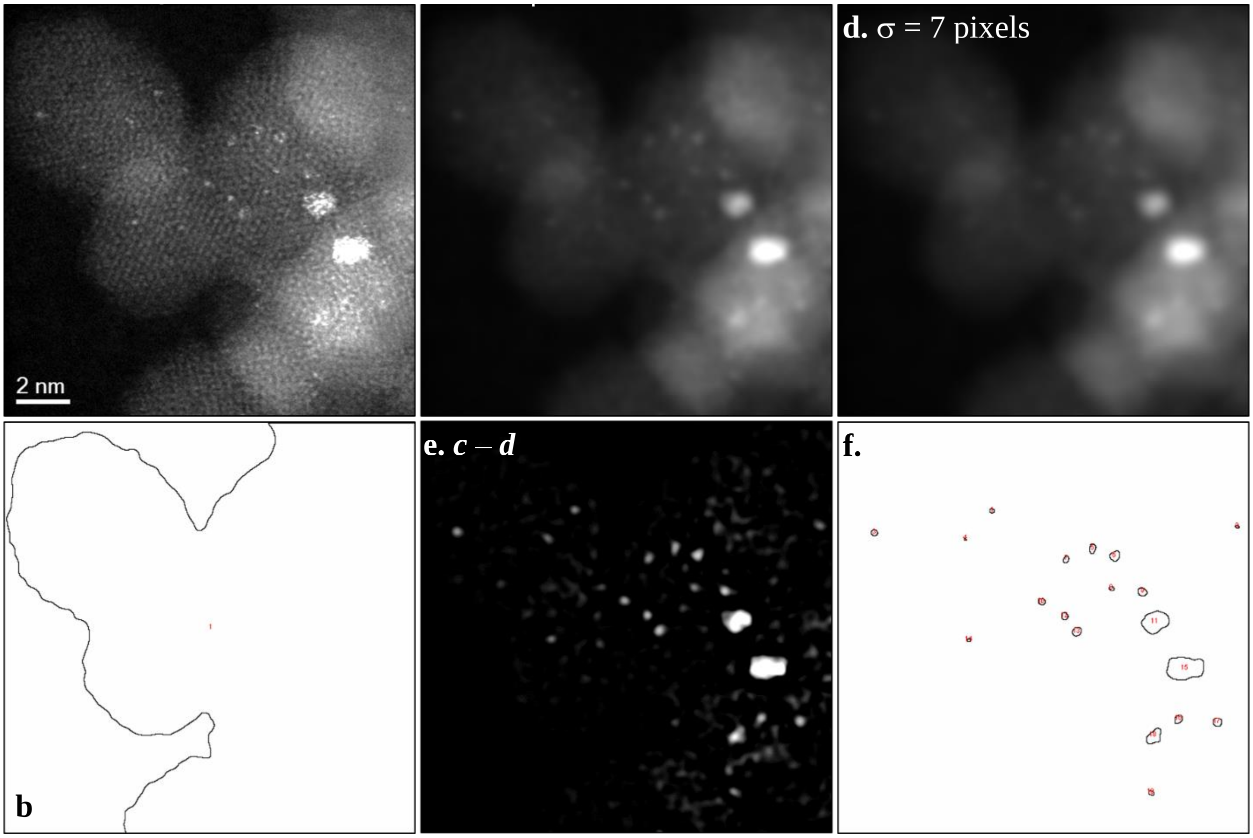

Supplementary Figure 4. Image processing sequence for analysis of particle size distribution. a. the original HAADF-STEM image; **b.** projected support area; **c.** image ***a*** convoluted with a 2D Gaussian with standard deviation σ = 5 pixels; **d.** image ***a.*** convoluted with a 2D Gaussian with σ = 7 pixels; **e.** image ***c*** subtracts image ***d***; **f.** The final particle recognition after removing the residual artefacts via global thresholding.

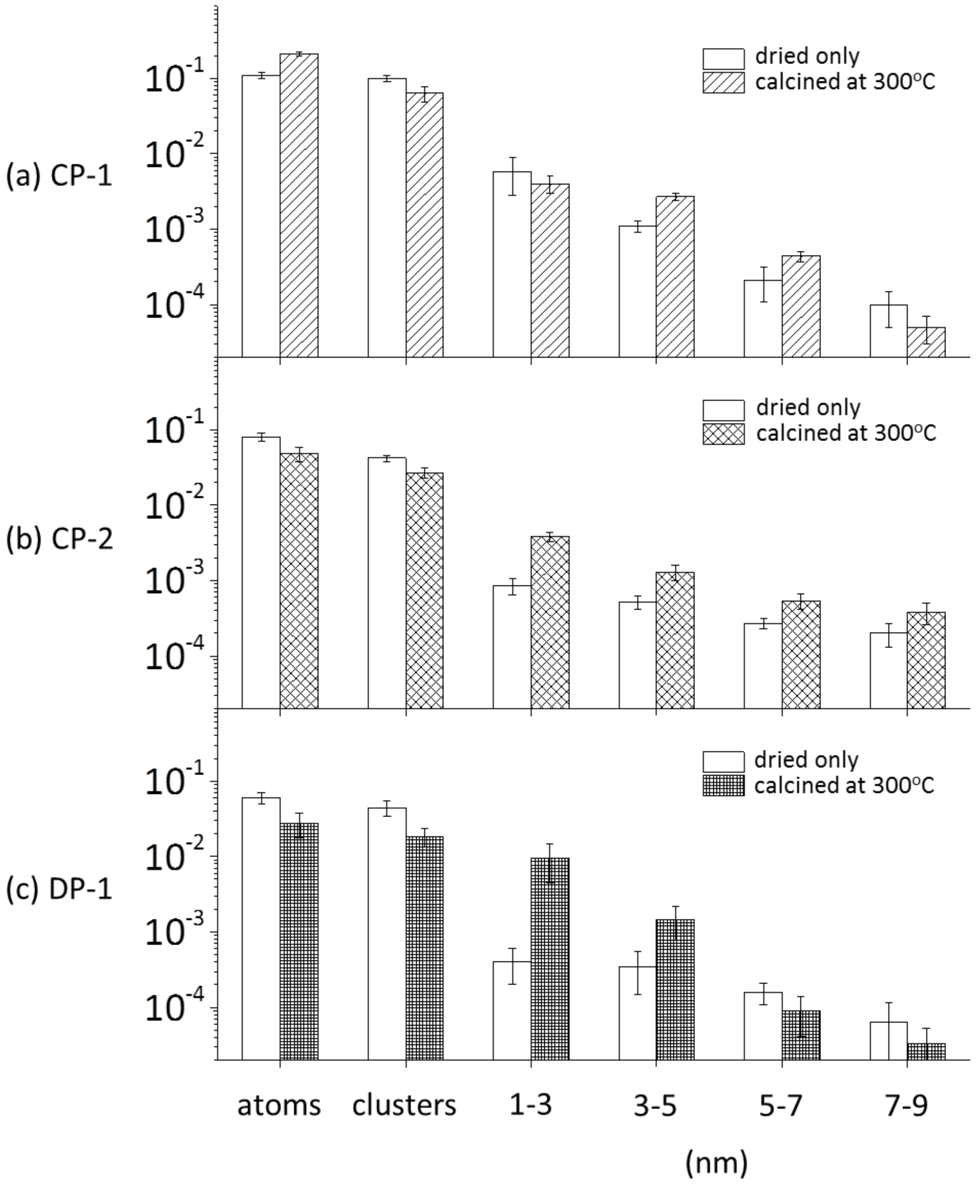

Supplementary Figure 5. Number density versus size histograms for the CP-1, CP-2 and DP-1 materials. Particle size distribution presented as number densities (log-scale) for the (a) CP-1 (acid-into-base), (b) CP-2 (base-into-acid) and (c) DP-1 (acid-into-base) catalysts at the 'dried only' and 'calcined' stages. Between 3000-5000 particles were counted for each sample. The error bars represent 90% confidence intervals.

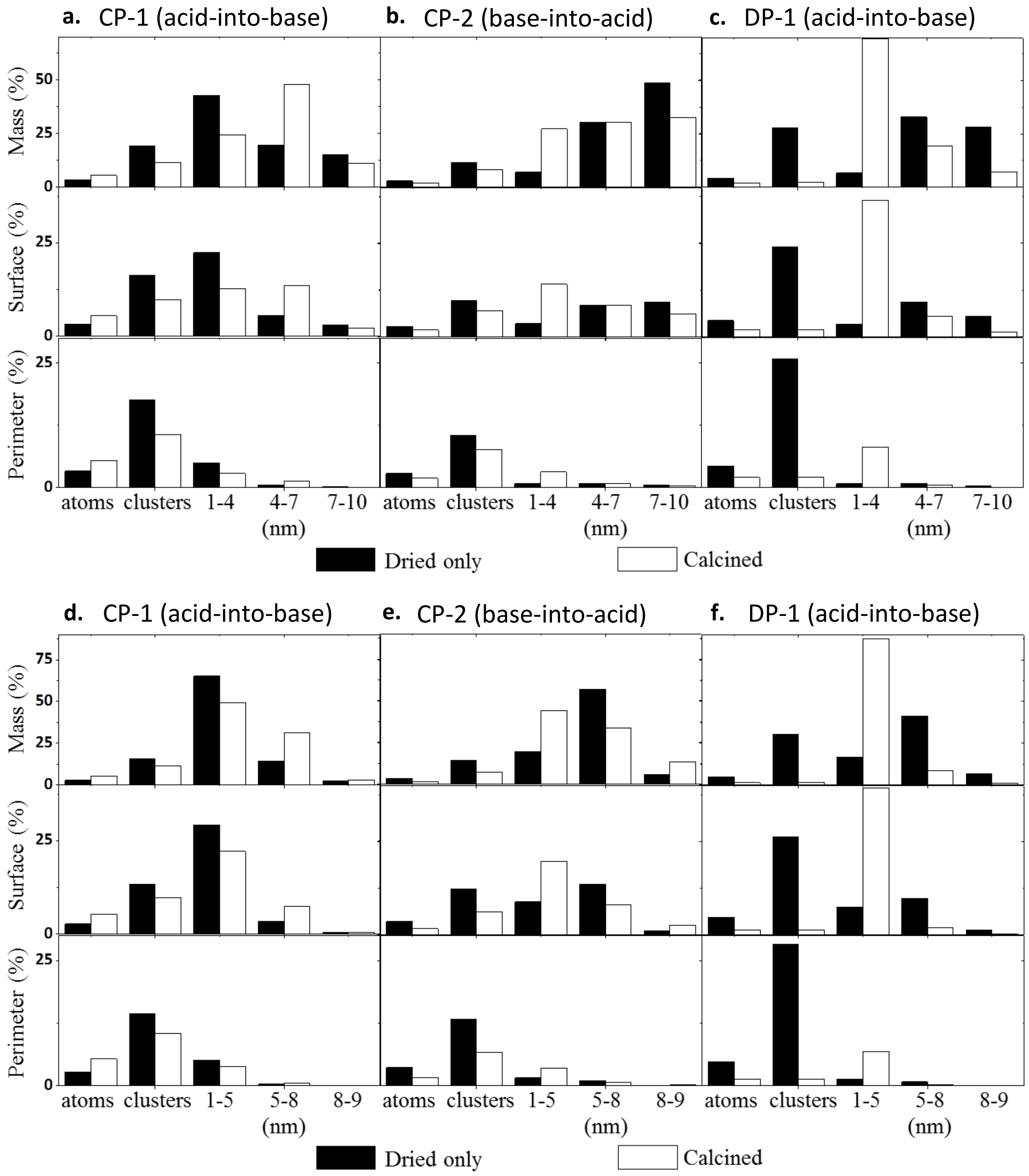

Supplementary Figure 6. Examples of mass fraction, surface fraction, and line perimeter distributions plotted with different binning sizes and upper end cut-off values. Mass, surface and perimeter fraction distributions presented with different binning intervals and upper cut-off values for the CP-1, CP-2 and DP-1 catalysts in the 'dried-only' and 'calcined' states. (**a-c**) Regular binning interval of 3 nm with an upper cut-off at 10 nm; (**d-f**) varying binning sizes with an upper cut-off at 9 nm.

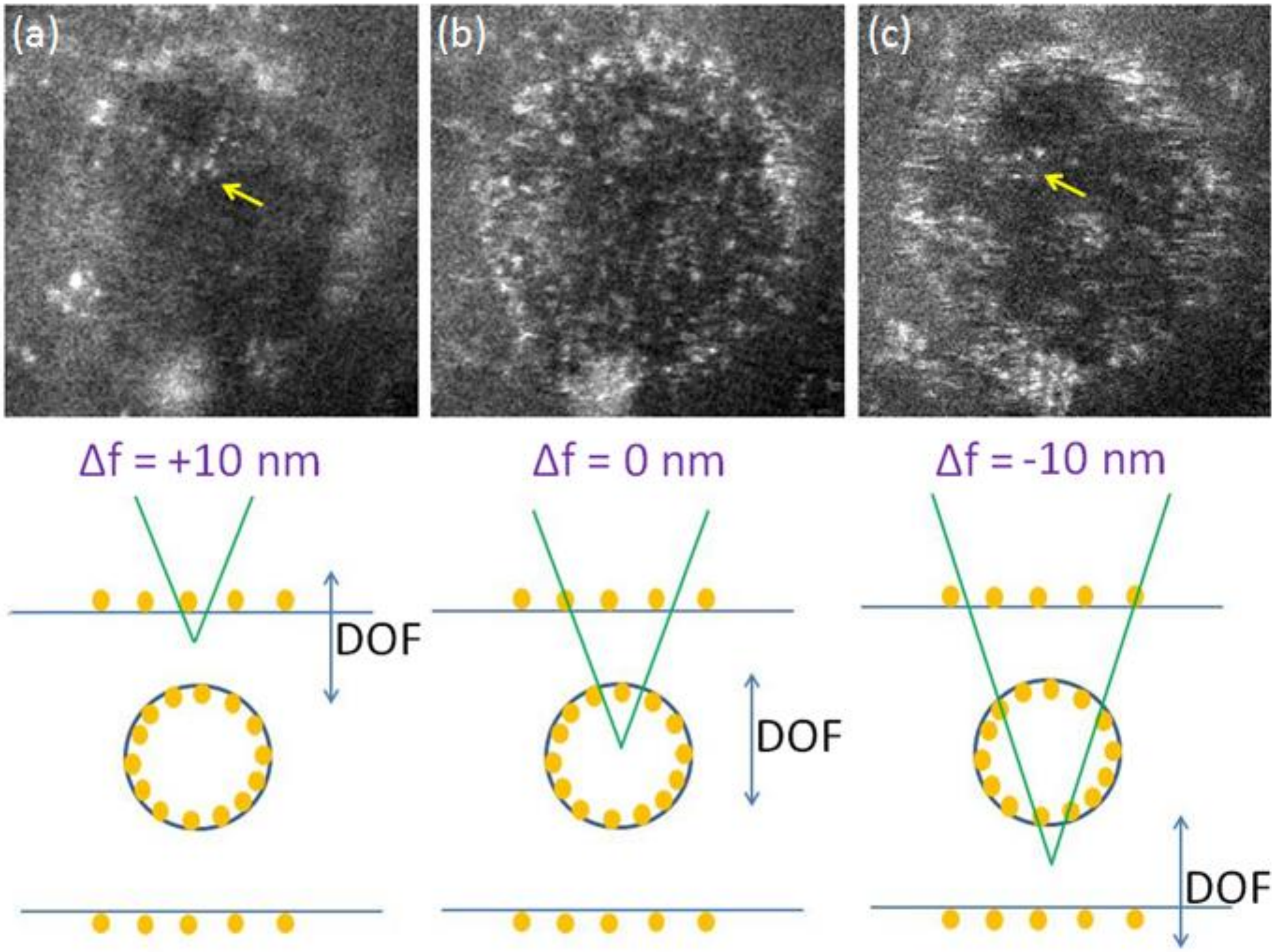

Supplementary Figure 7. Evidence showing Au atoms and sub-nm Au clusters are trapped inside the support of the CP-1 catalyst. Through focal series of STEM-HAADF images showing an internal pore in the CP-1 catalyst after calcination. DOF stands for “depth-of-field", which in the current circumstance is about 10 nm. The defocus values are calibrated relative to the middle image (b), given a nominal defocus value +10 nm (overfocus) and -10 nm (underfocus) from this position for images (a) and (c). For the image sequence (a) to (c), the electron beam is moving from the top surface towards the bottom surface. The Au atoms associated with the rim of the pore can be sharply imaged in (b). When these atoms on the rim are out of focus in (a) and (c), some additional Au atoms can still be found in focus projected inside the pore area (arrowed in white), suggesting that these atoms are either above or below the pore. They must therefore exist either on the top or bottom surface of the support, or on the internal pore surface, both indicating that this pore is fully located inside the support.

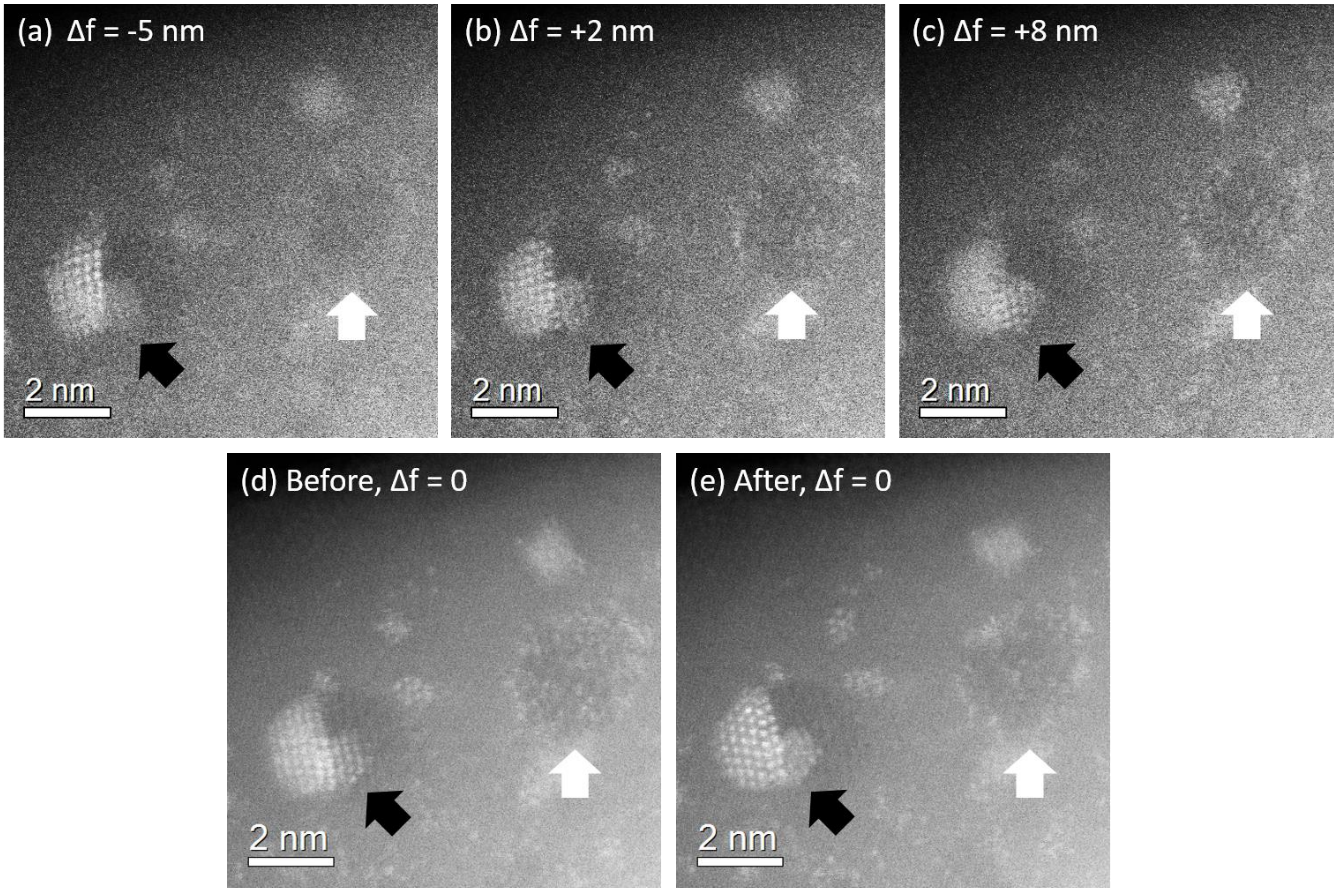

Supplementary Figure 8. Effect of electron beam irradiation on catalyst stability. Assessment of electron beam damage effects on the CP-1 catalyst sample. A through focal series of STEM-HAADF images (a)-(c) were taken using a 200kV Nion UltraSTEM. These show an internal pore (indicated by the white arrow) that is similar to that presented in Figure S6. The through-focal series of 100 frames (10nm field of view, 512*512 pixels, Δf=1 nm/frame) was taken with a probe current of 60pA and pixel dwell time of 2μs. The sample was estimated to have been subjected to a total electron dose of $2*10^8$ electrons/nm^2 during this entire process. The STEM-HAADF images (d) and (e) were taken before and after acquiring the through-focal series and the imaging conditions employed were identical except that a longer dwell time (16μs/pixel) was used. The electron beam did not induce significant agglomeration of the nanoparticles or sub-nm clusters. The internal pore (white arrow) retains its integrity, although some minor re-structuring/re-orientation of individual nanoparticles (indicated by the black arrow) did take place.

**Catalyst**	**Reaction Order between 20-60 °C**		**Activation Energy** (kJ mol^{-1})
	**CO**	**O_2**	
CP-1 dried only	1.0	0	26
CP-1 calcined	1.1	0	26
CP-2 dried only	1.2	0	30
CP-2 calcined	0.9	0	31

Supplementary Table 1. Results from kinetics measurements. Measured reaction orders with constant catalyst mass, 10 mg, and concentrations of CO (1 – 10 vol%) and O_2 (1 – 10 vol%) with a constant gas flow of 50 ml min^{-1}.

	Surface area / m^2 g^{-1}	
**Preparation Method**	**Dried only**	**Calcined**
CP-1 (acid-into-base)	255	138
CP-2 (base-into acid)	253	125
DP-1 (acid-into-base)	195	109
DP-2 (base-into acid)	235	161

Supplementary Table 2. Results from BET surface area measurements. BET surface areas of 5wt% Au/FeO_x catalysts prepared by the CP-1 and CP-2 methods and support materails for the DP-1 and DP-2 catalysts after drying at 120 ^oC and subsequent calcination at 300 °C for 3 h.

n (Shell No.)	Ideal Mackay model				Number used for calculation			
	d (diameter)	$N_{surface}$ (Full sphere)	N_{total} (Full sphere)	$N_{perimeter}$	Bin size	$N'_{surface}$ (half sphere)	N'_{total} (half sphere)	$N'_{perimeter}$
1	-	1	1	1	atoms	1	1	1
2	0.9 nm	12	13	6	Sub-nm clusters	6	7	6
3	2.0 nm	92	147	13	1-3 nm	46	74	13
7	4.3 nm	492	1415	29	3-5 nm	246	708	29
10	6.0 nm	1002	3871	40	5-7 nm	501	1935	40

Supplementary Table 3. Geometric model used to estimate the number of Au atoms associated with the exposed particle surface, entire particle volume and interfacial perimeter length as a function of Au particle size. The total number of exposed surface atoms, atoms per particle and interfacial periphery atoms estimated for Au particles of different sizes using the Mackay icosahedral model.[7] The left half of the table shows selected values of surface and total number of atoms from Mackay icosahedra having different diameters. The particle diameter d was estimated using the equation $d = (2n + 1) \cdot 0.288$ (nm), where n is the number of shells in the Mackay model; the total atoms per particle (N_{total}) was calculated using the equation $N_{total} = \frac{10}{3}n^3 + 5n^2 + \frac{11}{3}n + 1$; the total number of exposed surface atoms ($N_{surface}$) was calculated using the equation $N_{surface} = 10n^2 + 2$; the total number of atoms at the interfacial perimeter ($N_{perimeter}$) are estimated using the equation $N_{perimeter} = \pi d$/D, where D is the contribution of one atom in the perimeter, which can be estimated from the trivial case n = 2 and $N_{perimeter} = 6$. The Mackay icosahedron chosen to best represent each binning interval was that which had the closest value to the median value of the binning interval. The right half of the table are the actual numbers (N'_{total}, $N'_{surface}$ and $N'_{perimeter}$) based on 'hemispherical' Mackay icosahedron particles used to estimate the number of atoms in each size category. We assumed that all the particles are roughly hemi-spherical in shape, so therefore we use $N'_{total} = N_{total}/2$, $N'_{surface} = N'_{surface}/2$ and $N'_{perimeter} = N_{perimeter}$.

	Rate (mol CO s^{-1})	Sub-nm Au clusters	1-3 nm Au NPs
		(Surface fractions) Surface Au atoms (mol)	
**DP-1** (dried only)	3.1×10^{-7} (92% conv)	(28%) 7.1 $\times 10^{-7}$	(2.0%) 5.1 $\times 10^{-8}$
**DP-1** (calcined at 300°C)	1.7×10^{-7} (49% conv)	(5.3%) 1.4 $\times 10^{-7}$	(21%) 5.3 $\times 10^{-7}$
**Intrinsic reaction rate** (mol CO per mol Au per second)		**0.42**	**0.21**

Supplementary Table 4. Intrinsic rate estimation considering all surface atoms. Intrinsic reaction rates were estimated, assuming sub-nm Au clusters and 1-3 nm particles are the dominant active species and all surface atoms are active. *Testing conditions* – 100 ml min^{-1} flow of 0.5 vol% CO / air, 10 mg catalyst, 25 ºC.

	Rate (mol CO s^{-1})	Sub-nm Au clusters	1-3 nm Au NPs
		(Peripheral fractions)	Peripheral Au atoms (mol)
**DP-1** (dried only)	3.1×10^{-7} (92% conv)	(30%) 7.6×10^{-7}	(0.6%) 1.5×10^{-8}
**DP-1** (calcined at 300°C)	1.7×10^{-7} (49% conv)	(5.7%) 1.5×10^{-7}	(6.5%) 1.7×10^{-7}
**Intrinsic reaction rate** (mol CO per mol peripheral Au per second)		**0.4**	**0.6**

Supplementary Table 5. Intrinsic rate estimation considering only atoms at the perimeter. Intrinsic reaction rates were estimated, assuming sub-nm Au clusters and 1-3 nm particles are the dominant active species and only atoms at the perimeter are active. *Testing conditions* – 100 ml min^{-1} flow of 0.5 vol% CO / air, 10 mg catalyst, 25 ºC.

Supplementary Notes 1. Particle size measurements from the HAADF images.

The particle size was measured using Image J as illustrated in Supplementary Figure 4. The first step is to ascertain the projected area of the support: A global threshold is applied to convert the image to a binary image (- the pixels in the support area are labelled 1 and the pixels in the empty area are labelled 0) and then the support area equals the sum of the image. The second step is to measure the projected area of Au particles, including individual atoms, using their higher HAADF intensity as a criterion. Simple thresholding cannot be directly applied due to an uneven background caused by the support material having varying thickness in different regions. A two-step smoothing algorithm was applied to remove the effect of such a background: (i) the original image was first 2D Gaussian smoothed using a relatively small standard deviation σ (*e.g.* in Figure S3 (c), $\sigma = 5$ pixels) to just remove the lattice fringes from the support. (ii) A second 2D Gaussian smoothing operation was then applied with a slightly larger σ *e.g.* Supplementary Figure 4(d), $\sigma = 7$ pixels). The difference of images (c) and (d) is shown in Supplementary Figure 4(e), in which the Au particles and atoms are now clearly highlighted. Some residual artefacts can also be seen, which can be further removed by a thresholding method and the final results are shown in Supplementary Figure 4(f).

Supplementary Notes 2. Intrinsic reaction rate estimation.

It is possible to estimate the intrinsic activity for each type of Au species in the hierarchical ladder. The calculation has been performed on DP-1 catalyst, in which no Au species are expected to be buried inside the support. Since we only have two total activity data points (*i.e.* 'dried-only' and 'calcined' materials), we assume the *two* most dominant active species present to be (i) sub-nm Au clusters[8] and (ii) 1-3 nm Au NPs[8,9] based on previous studies.

The catalytic testing conditions employed were as follows: the reactant gas flow rate is 100 ml min^{-1} at 1 atm and room temperature (298 K), with CO concentration of 0.5 vol%. Using gas constant R = 82.05736 $(\text{ml atm K}^{-1}\,\text{mol}^{-1})$, the CO flow rate can be determined to be 3.4 x 10^{-7} mol s^{-1}. The conversion rates are 92% and 49%, which give the absolute rates to be 3.1 x 10^{-7} mol s^{-1} and 1.7 x 10^{-7} mol s^{-1} for the DP-1 'dried only' and DP-1 'calcined' materials respectively.

The next step was to calculate the number of surface atoms available on each type of Au species. Since we already have the data available for surface fraction, which is the fraction of total Au atoms that end up on the surface of Au species within size intervals, we only need to know the total amount of Au in the catalyst. The theoretical Au loading of the DP-1 catalyst was 5 wt%. As 0.01 g of catalyst was used in the catalysis testing experiment, this is equivalent to 0.0005 g Au or 2.54 x 10^{-6} mol. When qualitatively comparing activities between the dried only and calcined catalysts, we take the assumption that Au particles above certain size remain largely unchanged during the heat treatment and therefore we total amount of Au in the particles below that size (e.g. 7 nm) is approximately unchanged. This assumption is based on *in situ* studies by Allard *et al.*[10,11] However, when estimating the absolute reaction rate, further assumption that all the Au loading is in small particles is needed. In another word, we ignore the presence of all the larger particles > 7 nm, then the amount of Au atoms exposed on sub-nm Au clusters and 1-3 nm Au NPs in the 'dried-only' and calcined DP-1 catalysts can be calculated using surface and peripheral fractions from the PSD. The results are shown in Supplementary Tables 4 and 5 respectively. This approximate calculation tends to underestimate the absolute reaction rates, since the total mass of Au species < 7 nm must be less than the total Au loading due to the presence of larger particles.

Supplementary References.

1. Deng, W., De Jesus, J., Saltsburg, H. & Flytzani-Stephanopoulos, M. Low-content gold-ceria catalysts for the water-gas shift and preferential CO oxidation reactions. *Appl. Catal. A Gen.* **291,** 126–135 (2005).
2. Venugopal, A. & Scurrell, M. S. Low temperature reductive pretreatment of Au/Fe2O3 catalysts, TPR/TPO studies and behaviour in the water-gas shift reaction. *Appl. Catal. A Gen.* **258,** 241–249 (2004).
3. Guo, Y. *et al.* Uniform 2 nm gold nanoparticles supported on iron oxides as active catalysts for CO oxidation reaction: structure-activity relationship. *Nanoscale* **7,** 4920–4928 (2015).
4. Cui, H.-Z., Guo, Y., Wang, X., Jia, C.-J. & Si, R. Gold-Iron Oxide Catalyst for CO Oxidation: Effect of Support Structure. *Catalysts* **6,** (2016).
5. Martínez, J. J. *et al.* Hydrogenation of m-dinitrobenzene over Au catalysts on magnetic supports. *J. Mol. Catal. A Chem.* **383-384,** 31–37 (2014).
6. Neri, G., Visco, A. M., Galvagno, S., Donato, A. & Panzalorto, M. Au/Iron Oxide Catalysts: Temperature Programmed Reduction and X-Ray Diffraction Characterization. *Thermochim. Acta* **329,** 39–46 (1999).
7. Mackay, a. L. A dense non-crystallographic packing of equal spheres. *Acta Crystallogr.* **15,** 916–918 (1962).
8. Herzing, A. A., Kiely, C. J., Carley, A. F., Landon, P. & Hutchings, G. J. Identification of active gold nanoclusters on iron oxide supports for CO oxidation. *Science* **321,** 1331–5 (2008).
9. Liu, Y., Jia, C.-J., Yamasaki, J., Terasaki, O. & Schüth, F. Highly active iron oxide supported gold catalysts for CO oxidation: how small must the gold nanoparticles be? *Angew. Chem. Int. Ed. Engl.* **49,** 5771–5775 (2010).
10. Allard, L. F. *et al.* Evolution of gold structure during thermal treatment of AuFeOx catalysts revealed by aberration-corrected electron microscopy. *J. Electron Microsc. (Tokyo).* **58,** 199–212 (2009).
11. Allard, L. *et al.* Behavior of Au Species in Au/FeOx Catalysts as a Result of In-Situ Thermal Treatments, Characterized via Aberration-Corrected STEM Imaging. *Microsc. Microanal.* **15,** 1482 (2009).
